# Approximation of reliabilities for random-regression single-step genomic best linear unbiased predictor models

**DOI:** 10.3168/jdsc.2023-0513

**Published:** 2024-05-10

**Authors:** M. Bermann, I. Aguilar, A. Alvarez Munera, J. Bauer, J. Šplíchal, D. Lourenco, I. Misztal

**Affiliations:** 1 Department of Animal and Dairy Science, University of Georgia, Athens, GA 30602; 2 Instituto Nacional de Investigación Agropecuaria (INIA), 11500 Montevideo, Uruguay; 3 Czech Moravian Breeders' Corporation, Benešovská 123, 252 09 Hradištko, Czech Republic

## Abstract

Random-regression models (RRM) are used in national genetic evaluations for longitudinal traits. The outputs of RRM are an index based on random-regression coefficients and its reliability. The reliabilities are obtained from the inverse of the coefficient matrix of mixed model equations (MME). The reliabilities must be approximated for large datasets because it is impossible to invert the MME. There is no extensive literature on methods to approximate the reliabilities of RRM when genomic information is included by single-step GBLUP. We developed an algorithm to approximate such reliabilities. Our method combines the reliability of the index without genomic information with the reliability of a GBLUP model in terms of effective record contributions. We tested our algorithm in the 3-lactation model for milk yield from the Czech Republic. The data had 30 million test-day records, 2.5 million animals in the pedigree, and 54,000 genotyped animals. The correlation between our approximation and the reliabilities obtained from the inversion of the MME was 0.98, and the slope and intercept of the regression were 0.91 and 0.02, respectively. The elapsed time to approximate the reliabilities for the Czech data was 21 min.

## Highlights

• Reliabilities of genomic estimated breeding value in random-regression models must be approximated.
• We developed an efficient method to approximate them, including genomic information.
• The proposed method is accurate and efficient.

## Graphical Abstract

**Figure:**
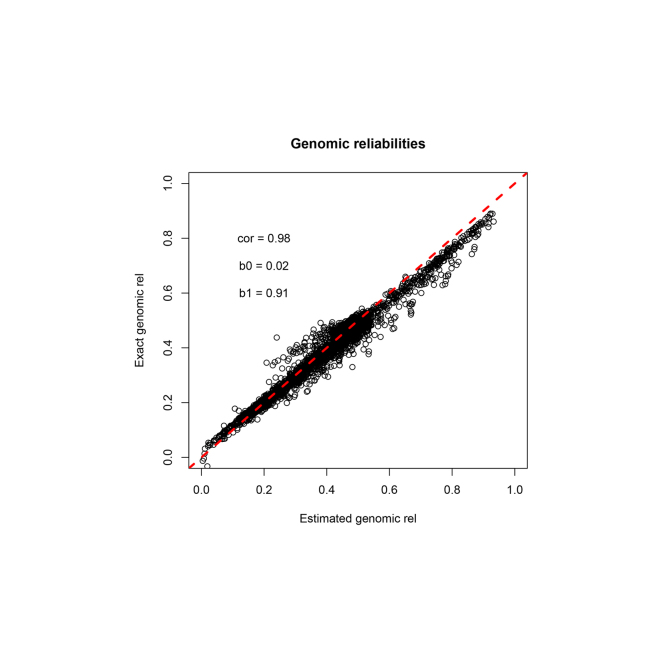
**Summary:** We developed a method to approximate the reliability of estimated breeding values in random-regression single-step genomic best linear unbiased predictor models. The proposed approximation is accurate as well as computationally efficient.

Random-regression models (**RRM**) are used for national dairy cattle genetic evaluations in many countries (Interbull, 2022). Typically, the traits for which RRM are fitted comprise production traits (milk, fat, and protein yield), SCS, conformation traits, and feed intake, among others (Schaeffer, 2004). Random-regression models have been used for traits in other species, such as growth in beef cattle (Meyer, 2005), residual feed intake in chickens (Emamgholi Begli et al., 2018), and pigs (Wang et al., 2022).

The output of a genetic evaluation with RRM is an EBV for a specific function of the additive genetic random-regression coefficients and its corresponding reliability. For example, for a model with 3 lactations, selection could be done by the average over the 3 lactations of the cumulative 305-d estimated breeding value. The reliability of an EBV in RRM is calculated from the prediction error variance (**PEV**) of the random-regression coefficients, which is obtained from the inverse of the coefficient matrix of mixed model equations (**MME**). In general, inverting the MME is computationally unfeasible for large datasets. For RRM, inverting the MME is even more complex because of the need of the prediction error covariances (**PEC**) between random-regression coefficients. Thus, approximation methods were developed for models without genomic information (Jamrozik et al., 2000; Liu et al., 2004; Tier and Meyer, 2004). Although they differ in implementation, the principle behind these methods is to weigh different information sources while processing the data and pedigree files twice. This approach does not imply large memory requirements or computing times thanks to the sparse structure of the inverse of the numerator relationship matrix (Henderson, 1976; Quaas, 1976).

The method of choice to include genomic information in RRM is single-step genomic best linear unbiased predictor (**ssGBLUP**; Aguilar et al., 2010; Oliveira et al., 2019a). Many studies successfully applied ssGBLUP with RRM in dairy cattle (e.g., Koivula et al., 2015; Kang et al., 2017; Oliveira et al., 2019b; Wang et al., 2022). Random-regression models with ssGBLUP might not be fully applied for routine genetic evaluations because no validated method exists to approximate the reliability of EBV for these models. Reliabilities for ssGBLUP based on RRM have been approximated by multiple-trait repeatability model reliabilities (Bauer et al., 2015). Nonetheless, a publication in the Interbull bulletin (Alkhoder et al., 2022) recognized the need for a method to approximate reliabilities for RRM with ssGBLUP.

Although different methods can approximate reliabilities in single- and multiple-trait ssGBLUP models (Liu et al., 2017; Edel et al., 2019; Bermann et al., 2021; Ben Zaabza et al., 2022), they are not easily extended to RRM. This is because of the need to calculate the PEC between different time points to obtain a final value of PEV and the need to process the information for the different random-regression coefficients altogether. Although the methods that do not consider genomic information approximate the PEC, they cannot be extended to account for genomic information because of the loss of sparsity of the model. Thus, this study aimed to develop an efficient method to approximate reliabilities for RRM with ssGBLUP.

In matrix notation, an animal random-regression multiple-trait model is

[1] y=Xb+Za+Qp+e,

where **y** is the vector of phenotypes, **b** is a vector containing sets of fixed effects independent and dependent on the time scale, **a** is the vector of additive genetic random-regression coefficients, **p** is the vector of permanent environmental effect, **e** is the error vector, **X** is an incidence matrix, and **Z** and **Q** are matrices of random-regression coefficients. It is assumed that

E[y]=Xb,

[2] $$Var\left(\begin{array}{c} a\\ p\\ e \end{array}\right)=\left(\begin{array}{ccc} A⊗G_{0} & 0 & 0\\ 0 & I⊗P_{0} & 0\\ 0 & 0 & I⊗R_{0} \end{array}\right),$$

where **A** is the numerator relationship matrix, **G**_0_ is the covariance matrix of the additive genetic random-regression coefficients, **P**_0_ is the covariance matrix of the permanent environmental random-regression coefficients, and **R**_0_ is the covariance of the error among traits. Given Equations 1 and 2, the MME for the RRM are

[3] $$\begin{array}{l} \left(\begin{array}{ccc} X^{\prime }R^{-1}X & X^{\prime }R^{-1}Z & X^{\prime }R^{-1}Q\\ Z\prime R^{-1}X & Z\prime R^{-1}Z+A^{-1}⊗G_{0}^{-1} & Z\prime R^{-1}Q\\ Q\prime R^{-1}X & Q\prime R^{-1}Z & Q\prime R^{-1}Q+I⊗P_{0}^{-1} \end{array}\right)\left(\begin{array}{c} \hat{b}\\ \hat{a}\\ \hat{p} \end{array}\right)=\\ \left(\begin{array}{c} X^{\prime }R^{-1}y\\ Z\prime R^{-1}y\\ Q\prime R^{-1}y \end{array}\right). \end{array}$$

For ssGBLUP, the numerator relationship matrix in Equation 2 is replaced by **H** as defined by Legarra et al. (2009), and the resulting MME are

[4] $$\begin{array}{l} \left(\begin{array}{ccc} X^{\prime }R^{-1}X & X^{\prime }R^{-1}Z & X^{\prime }R^{-1}Q\\ Z\prime R^{-1}X & Z\prime R^{-1}Z+H^{-1}⊗G_{0}^{-1} & Z\prime R^{-1}Q\\ Q\prime R^{-1}X & Q\prime R^{-1}Z & Q\prime R^{-1}Q+I⊗P_{0}^{-1} \end{array}\right)\left(\begin{array}{c} \hat{b}\\ \hat{a}\\ \hat{p} \end{array}\right)=\\ \left(\begin{array}{c} X^{\prime }R^{-1}y\\ Z\prime R^{-1}y\\ Q\prime R^{-1}y \end{array}\right), \end{array}$$

where the structure of **H**^−1^ is described in Aguilar et al. (2010).

To derive a general expression for the reliability of an animal's EBV, let **k**′ be a linear function defining the trait of interest, for example, 305 d for milk yield, and **a***_i_* be the vector of additive genetic random-regression coefficients for the animal *i*. Then, the breeding value of the *i*th animal is $u_{i}=k^{\prime }a_{i},$ and its respective EBV is ${\hat{u}}_{i}=k\prime {\hat{a}}_{i}.$ Note that ${\hat{u}}_{i}$ could be an index combining different traits. For instance, ${\hat{u}}_{i}$ could be a weighted average over different lactations of the cumulative 305 d EBV for milk yield. The reliability of ${\hat{u}}_{i}$ is

[5] $$rel_{i}=\frac{Var\left({\hat{u}}_{i}\right)}{Var\left(u_{i}\right)}=\frac{k^{\prime }C^{ii}k}{k\prime {\left(V⊗G_{0}\right)}_{ii}k},$$

where **V** can be either **A** or **H** and **C***^ii^* is the *i*th diagonal block of

[6] $$C={\left(V^{-1}⊗G_{0}^{-1}+Z\prime R^{-1}Z-\left[\begin{array}{cc} Z\prime R^{-1}X & Z\prime R^{-1}Q \end{array}\right]{\left[\begin{array}{cc} X^{\prime }R^{-1}X & X^{\prime }R^{-1}Q\\ Q\prime R^{-1}X & Q\prime R^{-1}Q+I⊗P_{0}^{-1} \end{array}\right]}^{-1}\left[\begin{array}{c} X^{\prime }R^{-1}Z\\ Q\prime R^{-1}Z \end{array}\right]\right)}^{-1}={\left(V^{-1}⊗G_{0}^{-1}+M\right)}^{-1}.$$

Equation 6 can be identified as the inverse of the coefficient matrix of a multiple-trait model whose covariance structure for the additive genetic random effects is $V⊗G_{0},$ and **M** is the matrix resulting from the absorption of all the effects except the additive genetic random effect. Evidently, with genomic information, one could treat Equation 6 as a multiple-trait model and approximate its reliabilities with an existing method. However, this would be computationally demanding. Instead, we assume there exists a single-trait linear model $y^{*}=X^{*}b^{*}+Z^{*}u^{*}+e^{*}$ such that $Var\left(u^{*}\right)=H,$ and the reliability of $u_{i}^{*}$ is

[7] $${rel}_{i}^{*}=1-\frac{W^{ii}}{H_{ii}}\approx rel_{i},$$

where **W***^ii^* is the *i*th diagonal element of $W=Var\left(u^{*}-{\hat{u}}^{*}\right)\;$ and can be approximated as

[8] $$W^{ii}\approx {\left(H^{-1}+D\right)}_{ii}^{-1},$$

where **D** is a diagonal matrix of weights. These weights should contain all the information from the model in Equation 1 and Equation 2 except for the relationships accounted for by **H**. This can be done by calculating reliabilities for ${\hat{u}}_{i}=k\prime {\hat{a}}_{i}$ without genomics (i.e., from the model of Equation 3) and back-solving them to obtain effective record contributions (**ERC**; Harris and Johnson, 2010; Bermann et al., 2021; Ben Zaabza et al., 2022).

Then, the procedure to obtain reliabilities for ${\hat{u}}_{i}$ in single-step RRM models is (P1):

1. For all of the animals, calculate rel*_i_* based on an RRM without genomic information using, for instance, the method of Tier and Meyer (2004).
2. Back-solve rel*_i_* to obtain **D**. Different back-solving methods will produce slightly different reliabilities. We chose to back-solve rel*_i_* by reversing the method of Harris and Johnson (1998). A detailed description of the algorithm is in the supplementary data of Bermann et al. (2021).
3. Approximate the single-trait ssGBLUP reliabilities $\left({rel}_{i}^{*}\right)$ following a standard method for ssGBLUP reliabilities (e.g., Liu et al., 2017; Edel et al., 2019). Namely, the main steps are as follows:
  a. Calculate reliabilities for a GBLUP or SNP-BLUP model using weights from **D**. The choice between GBLUP and SNP-BLUP would depend on the number of genotyped animals and the number of SNP.
  b. Obtain pedigree reliabilities for genotyped animals without considering information from nongenotyped animals. This step can be done with step 1 or by nullifying the weights in **D** corresponding to nongenotyped animals and recalculating reliabilities with the method of Harris and Johnson (1998) or Tier and Meyer (2004).
  c. Remove double counting in terms of effective record contributions and obtain final ssGBLUP reliabilities for genotyped animals.
  d. Propagate the information to nongenotyped animals.

A 3-lactation model from the Czech Republic was used for testing the proposed method. The full dataset had 30,366,184 test-day records for milk yield across 3 lactations. The number of records for each lactation was 13,991,590, 10,116,514, and 6,258,080, respectively. The pedigree included 2,518,681 animals from which 54,221 had available genotypes for 38,386 SNP. A total of 8,054 genotyped cows also had phenotypes. A reduced dataset was created to compare the approximate reliabilities against those obtained from the inversion of the coefficient matrix of MME. The reduced dataset had 111,494 test-day records, 44,582 animals in the pedigree, and 2,892 genotyped animals, from which 1,127 had phenotypes.

The proposed method was tested for the average of the 3 lactations of cumulative 305 d EBV based on comparing the approximated and the exact reliabilities (i.e., calculated from the inverse of the MME). The exact reliabilities were calculated based on the PEV of the additive random-regression coefficients obtained with the OPTION store_pev_pec from BLUPF90+ (Lourenco et al., 2022). The approximated reliabilities without genomic information (step 1 from P1) were obtained with the method of Tier and Meyer (2004) using ACCF90RR. Then, those reliabilities were given to ACCF90GS2 (Bermann et al., 2022) to perform steps 2 and 3 from P1.

Figure 1 shows the comparison between the reliabilities obtained by the inversion of the coefficient matrix of the MME and the approximated ones for the reduced dataset. The correlation between the exact and the estimated value was 0.98, whereas the slope and intercept of the regression of exact on estimated reliabilities were 0.91 and 0.02, respectively. For young-genotyped animals without records and progeny with records, the correlation was 0.93, and the slope and intercept were 0.94 and 0.06, respectively. These values are acceptable for routine evaluations and matched previous studies on approximation of reliabilities (Ben Zaabza et al., 2023). The observed underestimation in Figure 1 was because of young and inbred genotyped animals without records, for which the reliability without genomic information was underestimated. Figure 2 compares approximated reliabilities with and without genomics for the genotyped animals in the large dataset. It can be noticed that the gain in reliability is larger for young animals, which have low pedigree reliability. The animals that did not show an increase when adding genomics are nongenotyped animals, animals from commercial herds that are poorly connected with the population and have missing parents, or with diagonals of the genomic relationship matrix lower than the numerator relationship matrix. The approximation of all the single-step reliabilities for the large dataset took 21 min.

**Figure 1:**
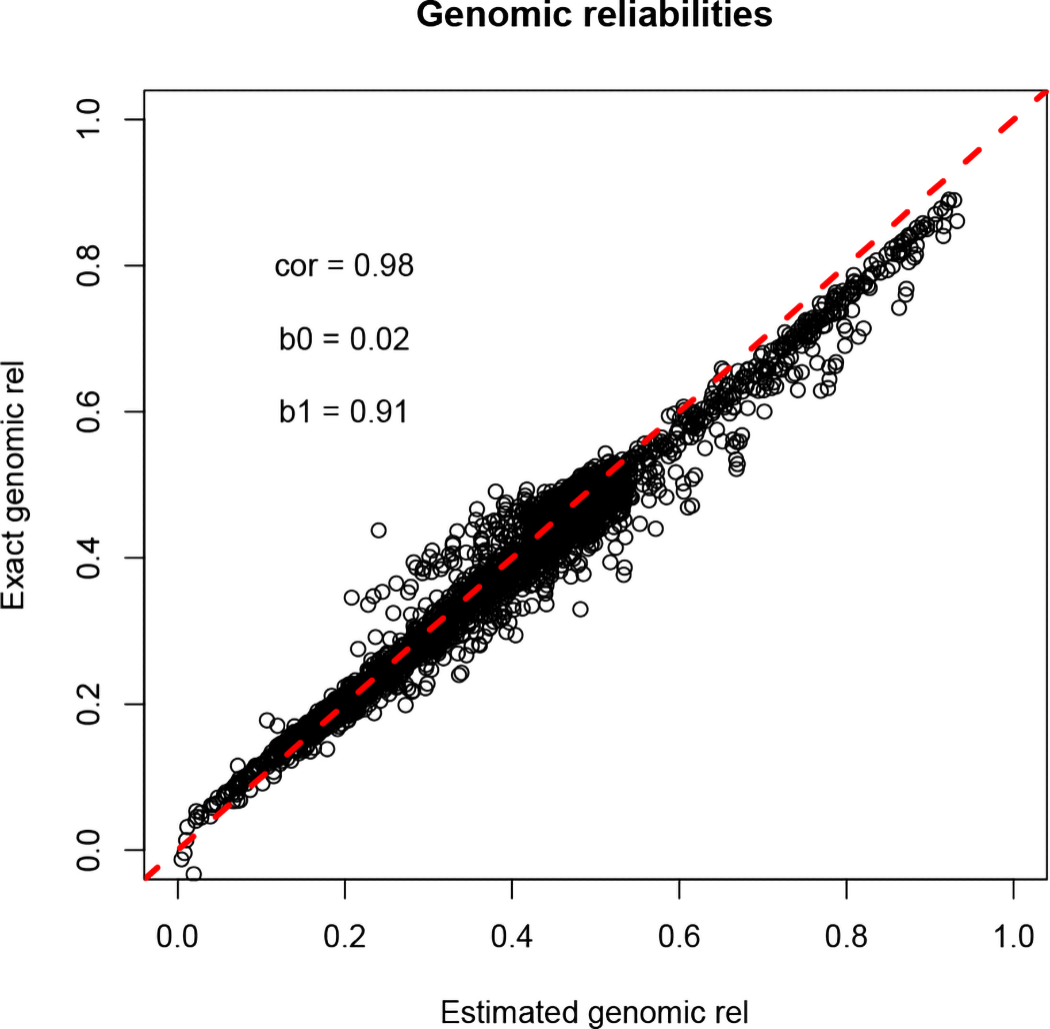
Comparison between the reliability (rel) obtained from the inverse of the mixed model equations and the approximated reliability for the small dataset. The correlation (cor), intercept (b0), and slope (b1) of the regression are reported. The red dashed line shows a line of slope equal to one and intercept equal to zero.

**Figure 2:**
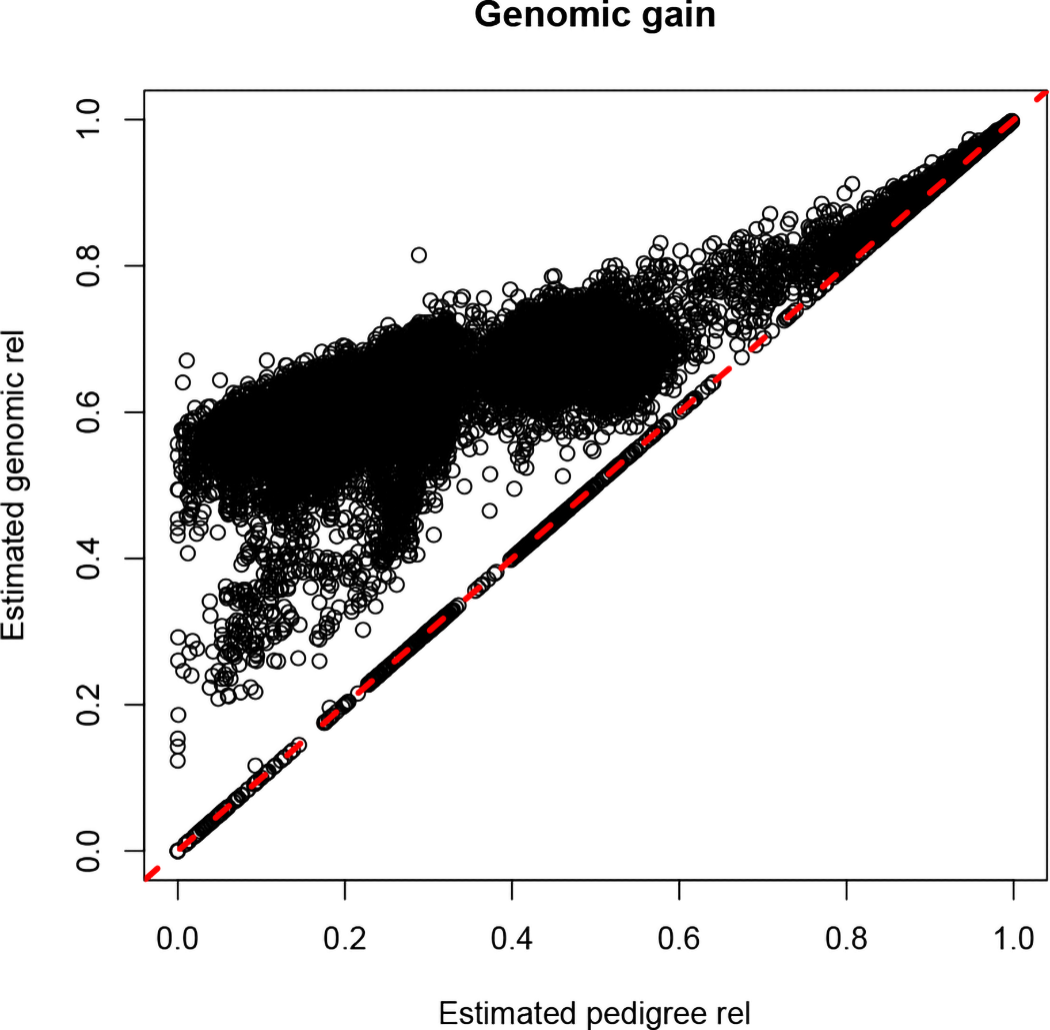
Comparison of approximated reliabilities (rel) with and without genomic information. The red dashed line shows a line of slope equal to one and intercept equal to zero.

The member countries of Interbull include MACE proofs in their official dairy evaluations to account for foreign information. Although the Czech Republic is a member of Interbull, neither Bauer et al. (2015) nor our group included MACE proofs in our study. The reason for this is that MACE proofs and test-day models are on a different scale, which adds another layer of complexity to the approximation of reliabilities. Boerner et al. (2023) presented a method to include MACE proofs directly into test-day models using de-regression and weights in terms of effective daughter contributions. However, they did not provide details on the approximation of reliabilities. Future research with the Czech data will include approximating reliabilities with foreign data.

Bauer et al. (2015) approximated the reliability of a single-step RRM using a multiple-trait repeatability model for Czech Holstein cows. The authors considered the first 3 lactations as separate traits and reported EBV obtained by averaging the values of 300 d of lactation over the 3 lactations. For each lactation, they approximated reliabilities for a single-trait model with a permanent environmental effect without genomic information using the method of Misztal et al. (1993). Then, they calculated ERC and obtained reliabilities for genotyped animals using the method of Misztal et al. (2013). After that, they adjusted reliabilities for nongenotyped animals. Finally, they calculated multiple-trait reliabilities with the method of Strabel et al. (2001) and used them to approximate the reliability of EBV. The authors did not compare the approximated reliabilities with those obtained by inversion of the MME. However, they observed that the reliability of young bulls without progeny increased on average by 0.02 when including genotypes for 2,236 animals. In our study, we observed an average increase of 0.42 for young bulls with a reference population of 54,221 animals. The main difference between the method of Bauer et al. (2015) and ours is that we do not approximate the RRM as a multiple-trait repeatability model. Instead, we aim to find a single-trait model with similar EBV reliabilities as the EBV calculated from an RRM. In other words, whereas Bauer et al. (2015) incorporate the genomic information while calculating the EBV reliability, we include the genomic information as a postprocessing step after obtaining the reliability of the index without genomic information.

In light of Equation 6, RRM are similar to multiple-trait models but with denser incidence matrices. Therefore, one could use a method to approximate reliabilities for ssGBLUP multiple-trait models in RRM (Bermann et al., 2021; Ben Zaabza et al., 2022). However, this would lead to a high computational cost, and how to approximate PEC between effects with genomics is unclear. The method of Bermann et al. (2021) could be implemented by treating each regressor as a single trait, approximating their reliabilities, and adjusting them for multiple traits as a postprocessing step. This would require calculating *n_trait_n_coef_* single-trait model reliabilities for all the animals, where *n_trait_* is the number of traits, and *n_coef_* is the number of additive genetic random-regression coefficients per animal. Since the most expensive task is to invert a single-trait GBLUP model, the computational complexity of this approach would be dominated by $O\left(n_{trait}n_{coef}n_{gen}^{3}\right),$ with *n_gen_* being the number of genotyped animals. For example, for the Czech Holstein dairy cattle evaluation, this implies calculating 12 single-trait reliabilities, which translates into inverting 12 times the genomic relationship matrix. The method of Ben Zaabza et al. (2022) can approximate the reliabilities for the additive random regressors altogether. However, this implies inverting a multiple-trait GBLUP model of size *n_trait_n_coef_n_gen_*, which translates into a complexity dominated by $O\left(n_{trait}^{3}n_{coef}^{3}n_{gen}^{3}\right).$ For the data used in this article, this implies inverting a matrix of size equal to 650,652 rows and columns. If a model does not include a residual polygenic effect, the multiple-trait GBLUP might be replaced with an equivalent SNP-BLUP model, leading to a complexity of $O\left[n_{trait}^{3}n_{coef}^{3}\;max\left(n_{snp}^{2}n_{gen},n_{snp}^{2}\right)\right],$ where *n_snp_* is the number of SNP. Since the running time for approximating reliabilities without genomic information goes from seconds to minutes, the difference in running times between the method of Bermann et al. (2021) and Ben Zaabza et al. (2022) would depend on how each method deals with genomic information. In other words, the difference between both methods would depend on whether the inversion of a *n_trait_n_coef_n_gen_* × *n_trait_n_coef_n_gen_* matrix is faster than inverting *n_trait_n_coef_* times a matrix of size *n_gen_* × *n_gen_*. This depends on the machine and level of parallelism allowed; however, the latter is usually faster. The method proposed in this article only requires calculating one single-trait reliability. The asymptotical complexity of the method is $O\left(n_{gen}^{3}\right).$ If the algorithm for proven and young (APY; Misztal 2016) is used, that complexity will drop up to $O\left[\max\left(n_{proven}^{3},\;n_{young}\;n_{proven}^{2}\right)\right],$ where *n_proven_* is the number of proven animals, which is usually between 5,000 and 35,000 animals (Pocrnic et al., 2016), and *n_young_* is the number of young animals, which is *n_young_* = *n_gen_* − *n_proven_*.

We developed a procedure for approximating the reliabilities of EBV for RRM with ssGBLUP. The proposed method is computationally efficient and accurate compared with the reliabilities obtained from the inversion of the MME. Thus, our approach can be implemented for routine genetic evaluations with RRM based on ssGBLUP.
